# Psychedelics and Consciousness: Expanding the Horizons of Mind and Therapy

**DOI:** 10.34133/research.0495

**Published:** 2024-10-04

**Authors:** Miyuan Zhang, Yibo Wang, Tian-Ming Gao, Xiaohui Wang

**Affiliations:** ^1^ Laboratory of Chemical Biology, Changchun Institute of Applied Chemistry, Chinese Academy of Sciences, Changchun 130022, China.; ^2^ School of Applied Chemistry and Engineering, University of Science and Technology of China, Hefei 230026, China.; ^3^ State Key Laboratory of Organ Failure Research, Key Laboratory of Psychiatric Disorders of Guangdong Province, Department of Neurobiology, Southern Medical University, Guangzhou 510515, China.

## Abstract

Psychedelics have long been recognized not only for their profound impact on human consciousness but also for their potential therapeutic applications. This perspective explores the multifaceted relationship between psychedelics and consciousness, emphasizing their capacity to alter sensory perceptions, disrupt self-referential thought processes, and catalyze profound spiritual and existential experiences. As research advances, psychedelics are being integrated into therapeutic settings, challenging existing psychiatric models and offering new insights into the complex nature of consciousness and mental health. This emerging paradigm marks the need for careful regulation and ethical considerations in the therapeutic use of psychedelics, promising a more holistic approach to mental health disorders.

What is the basis of consciousness? This is one of listed 125 critical scientific questions marking the *Science* magazine’s 125th anniversary in 2005 [1]. Nearly 2 decades later, it remains one of the most elusive and intriguing subjects in science today. Understanding complex systems often involves introducing disturbances and observing the outcomes. Meditation, although a profound tool for exploring and altering states of consciousness, is complex and challenging, with few practitioners able to achieve marked self-awareness or an expanded state of consciousness. Additionally, methods like transcranial magnetic stimulation and transcranial direct current stimulation offer noninvasive ways to stimulate the brain [2,3]. However, these techniques typically produce subtle effects that vary widely among individuals, further illustrating the challenges in consistently influencing consciousness.

Psychedelics, often referred to as “mind-manifesting” substances, have a profound impact on human consciousness and the potential to unlock doors to deep psychological insights and heightened sensory experiences. These effects can manifest as intensely vivid visions, emotional breakthroughs, and a sense of transcending ordinary reality. These substances, which include lysergic acid diethylamide, psilocybin, *N*,*N*-dimethyltryptamine, and mescaline, act primarily by stimulating serotonin receptors in the brain, precipitating a cascade of downstream signaling events. These signaling events further engage in brain function, notably altering perception, emotion, and cognition. Recent research has provided more detailed insights into these processes. For instance, psychedelics were demonstrated to promote neuroplasticity by activating intracellular signaling pathways associated with particular receptors, which enhances structural and functional neural plasticity [4–6]. This enhancement of neural plasticity is believed to contribute to the long-lasting therapeutic effects of psychedelics, as it facilitates the formation of new neural pathways and the reorganization of existing ones. Psychedelics also increase connectivity between different brain regions, which is thought to disrupt rigid patterns of thinking and behavior [7]. This increased connectivity allows for more flexible and creative problem-solving, as well as the integration of new perspectives and insights. Neuroimaging studies have consistently shown that psychedelics substantially reduce activity in the default mode network, a brain network crucial for self-referential thoughts and ego identity [8]. This reduction is correlated with experiences of ego dissolution, where the usual boundaries between the self and the external world become blurred, leading to feelings of unity with the universe or a profound sense of oneness. Historically used to explore and expand consciousness, psychedelics have recently gained acceptance in scientific research, marking a pivotal shift in recognizing their value in probing the mysteries of consciousness. This resurgence is bolstered by their controlled side effects, including low addiction potential and minimal harm, alongside a long history of use.

Consciousness and its alternations are central to understanding and treating neuropsychiatric diseases, as these disorders often involve disruptions in cognition, emotion, and behavior (elements that are influenced by consciousness). Individuals with neuropsychiatric disorders experience altered perceptions of reality, changes in thought processes, and difficulties in interacting with their environment. These symptoms are directly linked to modifications in brain chemistry and neural circuitry that are associated with consciousness.

Dr. Stanislav Grof, a pioneer in psychedelic therapy, notably remarked, “It does not seem to be an exaggeration to say that psychedelics, used responsibly and with proper caution, would be for psychiatry what the microscope is for biology and medicine or the telescope is for astronomy” [9]. This comparison highlights the unique ability of psychedelics to explore complex psychological processes that are typically inaccessible under normal circumstances. Psychedelics have demonstrated potential in reshaping consciousness by altering the brain’s activity patterns and connectivity. This can help patients acquire new perspectives and insights, leading to significant and lasting improvements in mental health.

Therapeutically, the psychedelics-induced ego dissolution and increased connectivity between different brain regions can help break the cycles of depressive thoughts, addiction behaviors, and other forms of mental rigidity. In clinical settings, psychedelics have shown efficacy in treating a range of neuropsychiatric conditions, such as depression, post-traumatic stress disorder, anxiety, and addiction, particularly in cases where conventional treatments like selective serotonin reuptake inhibitors (SSRIs) have failed [10–14]. While some studies suggest that psychedelics may not be significantly more effective than SSRIs in some cases, they offer unique benefits in promoting psychological insights and personal growth, which can be particularly valuable in treatment-resistant cases without significant delayed therapeutic effects [10,15]. In fact, the subjective experiences induced by psychedelics are closely correlated with their therapeutic effects.

The therapeutic use of psychedelics has been linked to increased neural plasticity, enhancing the brain’s ability to form new connections and reorganize existing ones [5]. From a predictive coding perspective, psychedelics may alleviate rigid thinking patterns and reduced cognitive flexibility by disrupting maladaptive predictive models that the brain has formed. This disruption allows for the reassessment and updating of these models, potentially leading to more adaptive and flexible cognitive processes [7]. This aspect is particularly beneficial for patients with neuropsychiatric diseases, who often suffer from rigid thinking patterns and reduced cognitive flexibility. Psychedelics promote a more flexible state of consciousness, potentially alleviating symptoms and enhancing the quality of life for those affected by these disorders. Unlike traditional neuropsychiatric medications, psychedelics do not require repeated or periodic dosing. A single or few doses can maintain therapeutic effects for extended periods, ranging from several months to years, or even a lifetime [15,16]. This could revolutionize treatment approaches, particularly as ongoing research continues to validate psychedelics’ role in enhancing our comprehension of consciousness and expanding treatment options for neuropsychiatric diseases.

However, the integration of psychedelics into therapeutic settings is not without its challenges and ethical considerations. The legality of these substances varies significantly across different regions, with many countries still classifying them as controlled substances. Ethically, the profound effects of psychedelics need careful and responsible usage under professional supervision to mitigate potential risks, including psychological distress or exacerbation of existing mental health issues. It is imperative to establish rigorous regulatory frameworks and informed guidelines to ensure patient safety and the responsible use of these substances. Informed consent is particularly crucial, given the intensity and unpredictability of psychedelic experiences. Furthermore, the potential for misuse requires the need for controlled environments where therapeutic use can be monitored and managed effectively. Addressing these ethical considerations is essential for the safe and effective integration of psychedelics into psychiatric care. Moreover, recent studies suggest that the therapeutic benefits of psychedelics may not necessarily depend on their hallucinogenic effects. For instance, nonhallucinogenic psychedelic analogs were identified to exhibit antidepressant-like effects in animal models [17,18]. Similarly, an engineered biosensor was developed to discover psychedelic-inspired compounds that provide therapeutic benefits without hallucinations [19]. These advances open up new avenues for the development of psychedelic-based treatments that minimize the risks associated with hallucinogenic experiences. It is important to note that these findings are primarily based on rodent models, which have limitations in fully replicating the complexity of human depression and its treatment [20].

In conclusion, the ongoing research into psychedelics and consciousness is not only reshaping our understanding of the human mind but also pioneering new methods for treating complex psychiatric disorders. As these substances challenge and expand the boundaries of traditional neuroscientific and psychiatric approaches, they open up innovative avenues for therapy and personal introspection. The unique pharmacological effects of psychedelics enhance cognitive flexibility, promote creative problem-solving, and deepen our understanding of consciousness, thereby offering promising therapeutic benefits. This exploration is instrumental in developing more holistic and effective mental health interventions that are finely attuned to the complex nature of human consciousness. Ultimately, psychedelics serve as critical tools in advancing neuropsychopharmacology and brain science, driving forward our comprehension of the mind and potentially transforming approaches to mental health care to better address the underlying experiences and needs of those with neuropsychiatric disorders.
